# Progress in Pediatrics

**Published:** 1901-05-04

**Authors:** 


### Progress in Pediatrics.

**Recurrent Vomiting in Children.**—Several cases of this affection are related by Dr. Crozer Griffith [1] and the literature of the subject is reviewed. It has also been described under the names of "fitful" or "cyclic vomiting." A similar condition is met with in adults, quite apart from any organic disease, which closely simulates the gastric crises of tabes, but which seems to be distinguished from such vomiting in children by the presence of severe abdominal pain. In young children an attack usually sets in with but slight prodomal symptoms in the form of malaise, furring of the tongue, and possibly constipation, and lasts from two to five days or longer. Vomiting is the first marked symptom, and is obstinate and frequent. The appetite is completely lost, the tongue is furred, thirst is intense, intractable constipation with retraction of the abdomen is usually present, and there may be more or less fever. The state of the child soon becomes one of alarming collapse, with sighing and irregular respiration, extreme restlessness, and scanty urine, which may contain indican or albumin and casts. In some cases death occurs from exhaustion, and post-mortem examination has failed to reveal any lesion to which the symptoms could be ascribed. In others, convalescence sets in after cessation of the vomiting, and recovery is extremely rapid. As a rule the attacks come on at irregular intervals of, it may be, three to six months, or longer. Dr. Griffith is inclined to regard the condition as one of toxæmia, probably not uric acid, but some toxalbumin allied to it. Treatment does not seem to be of much avail, although in one case recorded by the author the subcutaneous administration of morphine seemed to be of service, but recovery usually follows even although the patient may be in a condition of extreme collapse.

**Cyclic Albuminuria of Adolescence.**—Dr. Dauchez [2] reports two cases of this affection, which he traces in some cases to a gouty diathesis and in others to infective disease. Our patient was a girl of thirteen years, of markedly gouty parentage, who for a period of ten months had evening albuminuria, with occasional intermissions. Curiously enough he states that during the menstrual epochs evening albuminuria was present, whereas some years ago Dr. Teissier, of Paris, found that cyclic albuminuria did not occur during menstruation. Even after the albuminuria had ceased entirely for four months at ordinary times, it recurred with every monthly period. He considers that prolonged treatment, chiefly of a dietetic nature and addressed to the gouty dyscrasia, is necessary, and that a cure should have taken place before marriage is advisable.

**Neuroses of Childhood.**—In the Ingleby Lectures of last year Dr. O. J. Kauffmann [3] discussed the commoner neuroses of childhood, under which term he includes chorea, saltatory or salaam spasm, tetany, night terrors and dreaming, migraine, and epilepsy. His chief attempt is to show that in all these affections there is an underlying condition of toxæmia, and that while the poison of definite infective disease is present in a certain number of cases, in the vast majority self-infection takes place from the gastro-intestinal tract. Taking, as an example, chorea, he finds that the sufferers are small, ill-clad, and ill-fed; that they have often furred tongues and vascular stigmata on the cheeks; that a history of indigestion and constipation will usually be elicited; and that indican is frequently present in the urine. In the treatment of all such cases attention must be directed to the prevention of overfeeding, of unsuitable food, of a catarrhal condition of the stomach or small intestine, and of constipation. The underlying nervous instability will also require treatment, medicinal and otherwise, which varies with the special symptoms.

**Analgesia by Spinal Injection.**—This method has been employed in the case of children by Dr. W. S. Bambridge,[4] who reports favourably after an experience of seven cases including hip disease, umbilical hernia, and congenital talipes. The skin at the site of puncture was first frozen by an ethyl chloride spray and then the needle was introduced into the spinal canal, care being taken to avoid the central part. The injection was given slowly, lasting from 1½ to 2½ minutes. Before injection as much cerebro-spinal fluid is allowed to run out as it is proposed to insert. Often the first evidence that the cocaine was taking effect was some dilatation of the pupil or a slight nausea. During the operation a nurse known to the patient was close at hand, but no blindfolding was employed, and the instruments were kept out of sight as much as possible. In all the cases any after-effects of an unpleasant nature were merely temporary.

**Pathogenesis of Rickets.**—Writing on this subject Dr. Eric Pritchard[5] traces in certain cases the onset of rickets to persistent over-feeding with consequent development of lactic acid. The various steps in the process are thus traced:—The infant becomes fat, and by thus storing the surplus food does not manifestly suffer. At times vomiting and diarrhœa come on and are Nature's method of defence against the over-feeding. But when patent and predigested foods are used to meet this so-called weakness of digestion they, being extremely diffusible and bland, may be absorbed and will get stored up in the form of glycogen in the liver, muscles, and other tissues. So far there have been no manifest symptoms of rickets, but when the physiological limit of fat deposition is reached, and the supply of oxygen is not sufficient to deal with the excess of carbohydrates, then complete metabolism does not take place and lactic acid is formed. The lactic acid will be partly neutralised by the alkaline salts of the blood, and distributed for further oxidation to the skin, mucous membranes, liver, spleen, and thymus gland. As long as these structures are able to meet this demand on them no serious symptoms may appear, but, when they fail to do so, lactic acid and carbonic acid will accumulate in the system, which will become partially paralysed by the products of its own metabolism. Such is the clinical picture drawn by the author of the development of rickets in an infant saturated with carbohydrates, and he maintains that the condition can be cured merely by reducing the amount of carbohydrates to physiological proportions.

[1] Amer. Jour. Med. Sci., Nov. 1900. [2] Jour. des. Sci. Med. de Lille, Oct. 6, 1900. [3] The Birm. Med. Rev., Aug. 1900. [4] Med. Rec., Dec. 15, 1900. [5] Arch. of Pediat., Feb. 1901.

